# Use of symptom-focused oncological cancer therapies in hospices: a retrospective analysis

**DOI:** 10.1186/s12904-020-00648-4

**Published:** 2020-09-12

**Authors:** Ulrich Kaiser, Ursula Vehling-Kaiser, Fabian Kück, Nicolae-Catalin Mechie, Ana Hoffmann, Florian Kaiser

**Affiliations:** 1 University Hospital Regensburg, Clinic and Polyclinic for Internal Medicine III, Regensburg, Germany; 2 VK&K Studien GbR, Landshut, Germany; 3 Department of Medical Statistics, University Medical Center Göttingen, Göttingen, Germany; 4 University Medicine Göttingen, Clinic for Gastroenterology and Gastrointestinal Oncology, Göttingen, Germany; 5 University Medicine Göttingen, Clinic for Haematology and Medical Oncology, Robert-Koch-Straße 40, 37075 Göttingen, Germany

**Keywords:** Symptom-focused oncological cancer therapy, Hospice, Symptom relief

## Abstract

**Background:**

There is controversy regarding the practical implementation of symptom-focused oncological cancer therapies to hospice residents. In this study, we aim to analyse the use and indication of supportive-oncological cancer therapies in hospices.

**Methods:**

We conducted a retrospective survey of all residents of two hospice centres in the government district of Lower Bavaria, Germany. Hospice 1 (H1) was a member of an oncological–palliative medical network, and hospice 2 (H2) was independently organized. The evaluation period was the first 40 months after the opening of the respective hospice care centre. Demographical and epidemiological data as well as indications and type of supportive-oncological cancer therapies were recorded. A descriptive analysis and statistical tests were performed.

**Results:**

Of the 706 residents, 645 had an underlying malignant disease. The average age was 72 years and the mean residence time was 28 days. The most frequent cancer types were gastrointestinal cancers, gynaecological cancers and bronchial carcinomas. Overall 39 residents (33 in H1 and 6 in H2, *p* < 0.01) received symptom-focused oncological cancer therapy. The average age of these residents was 68 years, and the mean residence time was 55 days. The most common therapeutic indications were dyspnoea and pain. The most common symptom-focused oncological cancer therapies were bisphosphonates, transfusions (erythrocyte- and platelet- concentrates), radiotherapy and anti-proliferative drugs (chemotherapy, anti-hormonal- and targeted- therapies). Patients with therapy lived significantly longer than patients without therapy (*p* < 0.01).

**Conclusions:**

Symptom-focused oncological cancer therapies can be implemented in hospices; however, their implementation seems to require certain structural and organizational prerequisites as well as careful patient selection. As a palliative medical approach, the focus is to ameliorate the symptoms and not prolong life. Symptom-focused oncology treatment could be a further and important part for the therapy of hospice patients in the future.

## Background

Introductory case report: An elderly man (80 years old) was admitted to the hospice because of advanced bronchial carcinoma. He suffered from dyspnoea and weakness due to anaemia (Hb 7–8 g/dl, no pleura effusions, no stridor) during physical strain, but he managed the daily activities in the hospice. During his daughter’s wedding, where > 300 guests were invited, he wanted to start the wedding reception with a bridal dance. Two erythrocyte concentrates were transfused. As a result the dyspnea and the weakness were improved. The dance was a success; father and daughter were overjoyed, and the guests applauded. A few days later the resident died in the hospice centre. Thus, symptom-focused cancer therapy at the end of life was the subject of this retrospective analysis.

The use of palliative care for the treatment of critically ill patients is increasing. The early use of palliative care during metastatic stages or for previously incurable cancers is becoming increasingly important, particularly in the field of cancer therapy [1–4]. Palliative care focuses on measures to improve the quality of life [2, 3] and is often used during early stages of cancer parallel to oncological therapies [5]. In contrast, the implementation of cancer therapies in patients with advanced stages of diseases, especially in hospices, is controversial. However, cancer therapies should be differentiated into oncological therapies, primarily aimed at prolonging life or preventing further cancer growth, and symptom-focused cancer therapies, focused primarily on maintaining or improving the quality of life. Patients, even in advanced disease stages, can benefit from modified symptom-oriented cancer therapies [6–13]. A parallel application of cancer therapies and hospice care can be useful [14, 15] and even decrease the use of more aggressive therapies [16]. Examples of symptom-controlling oncological therapies are chemotherapy, antihormonal therapies, radiotherapy, bisphosphonates, or transfusions [8–13, 15, 17, 18], which are used in cancer-associated conditions such as symptomatic anaemia/thrombocytopenia, pain, symptomatic bone metastases, or difficult to stop local bleeding. However, the use of cancer therapies must be critically evaluated, particularly during the late stages of life, to prevent a reduction in the quality of life or excessive therapy [19–22]. The more a cancer progresses, the more difficult it becomes to provide the indication for cancer therapies. In particular, the residents of hospices who are in the terminal stage of their illness must always be given special consideration [19]. The decision whether to provide palliative care, hospice care, or further cancer therapy for a patient with a highly advanced stage disease is often very difficult [23]. However, the omission of life-sustaining therapies alone does not seem to be optimum to identify patients who benefit from a hospice [24]. There are indications, that streamlined concepts combining disease-specific therapies and hospice care have advantages over a strict “either/or” concept [25–27].

## Methods

The aim of the study was to determine to what extent and with what indication oncological therapies are still used in hospices of a governmental district in the State of Bavaria, Germany. The basis for a further discourse is to be established in this way.

Therefore, in the sense of a cluster sample, we performed a retrospective analysis in all hospices in Lower Bavaria (*n* = 2). This method was selected for practical and economic reasons. The evaluation period was the first 40 months after opening [Hospice 1 (H1): 09/2013 to 12/2016; Hospice 2 (H2): 07/2015 to 10/2018]. For these periods, all residents of the two hospices were included in the study without exception in a first analysis step. The date of birth, age at admission, gender, date of admission, date of death, date of discharge (if applicable) and the underlying main diagnosis were recorded for all residents. In a second examination step, all patients with a malignant haematological or oncological disease as the main diagnosis were included in the further analysis. Patients with a non-malignant main diagnosis were excluded. Further inclusion and exclusion criteria (e.g. age, clinical course and cause of death) did not exist. In this second analysis step, the use and, if available, the indication and type of cancer-specific therapies were included.

Cancer-specific therapy was defined as haematological and oncological therapies (chemotherapy, targeted and anti-hormonal therapies, bisphosphonates, radiotherapy, platelet and erythrocyte transfusions; hereinafter referred to as symptom-focused oncological therapies) that are commonly used to treat malignant diseases. Because checkpoint inhibitors were only approved for individual indications or were not yet approved during the evaluation period, they were not included in the evaluation. Palliative-supportive or general internal therapies such as anti-emetics, pain medication or antibiotics were not considered. Furthermore, demographic and structural data of both hospices were documented.

Medical personnel trained in oncology/palliative medicine and experienced in scientific data collection were assigned to carry out the documentation to ensure a high level of content and quality of the data collected. Data was obtained from the patient files of the respective hospices. For this purpose, a data entry form was created in Microsoft Excel 2010, which was used in both hospices. Content controls were included as part of the data evaluation. In the event of obvious discrepancies, these were checked again using the original documents and corrected if necessary. To guarantee the anonymity of the hospice residents, a pseudonymized procedure was selected, in which each resident was assigned a number for further evaluation.

Both the hospice centres had a capacity of 10 beds, with comparable populations of the respective county (H1, 158,025 inhabitants; H2, 119,075 inhabitants). H1, the member of an oncological–palliative medical network, is certified by the European Society for Medical Oncology (ESMO) and located in the immediate vicinity of a hospital and haematological/oncological outpatient clinic. Furthermore, regular hospice conferences are held with palliative care physicians, haematologists/oncologists, general practitioners and nursing staff of the hospice, during which the indications for symptom-focused oncological therapies are discussed on interdisciplinary basis. In H2 hospice conferences have not yet been established. H2 is independently organized. Medical care in H1 is provided by general practitioners, haematologists/oncologists and palliative care physicians, whereas in H2 it is provided by general practitioners and palliative care physicians.

For the comparison of the two hospices and the comparison of patients who received an oncological therapy and patients who did not, we applied Fisher’s exact test for binary variables and the Wilcoxon rank sum test with continuity correction for numerical variables since a visual inspection showed that these are not approximately normally distributed. In order to analyse survival times, we generated Kaplan–Meier curves and compared them by performing log-rank tests. Moreover, predictors were identified using multivariate Cox regression models. Due to the exploratory nature of this study, no adjustment for multiple testing was applied. The significance level was set to alpha = 5% for all statistical tests. All analyses were performed with the statistic software R (version 3.4.0 [28];) using the R-packages survminer (version 0.4.4 [29];) and survival (version 2.41.3 [30];) for Kaplan-Meier curves and Cox regression models.

According to the Ethics Committee Munich, an ethical approval was not required for this study.

## Results

### Entirety of patients with an underlying malignant disease

**Fig. 1:**
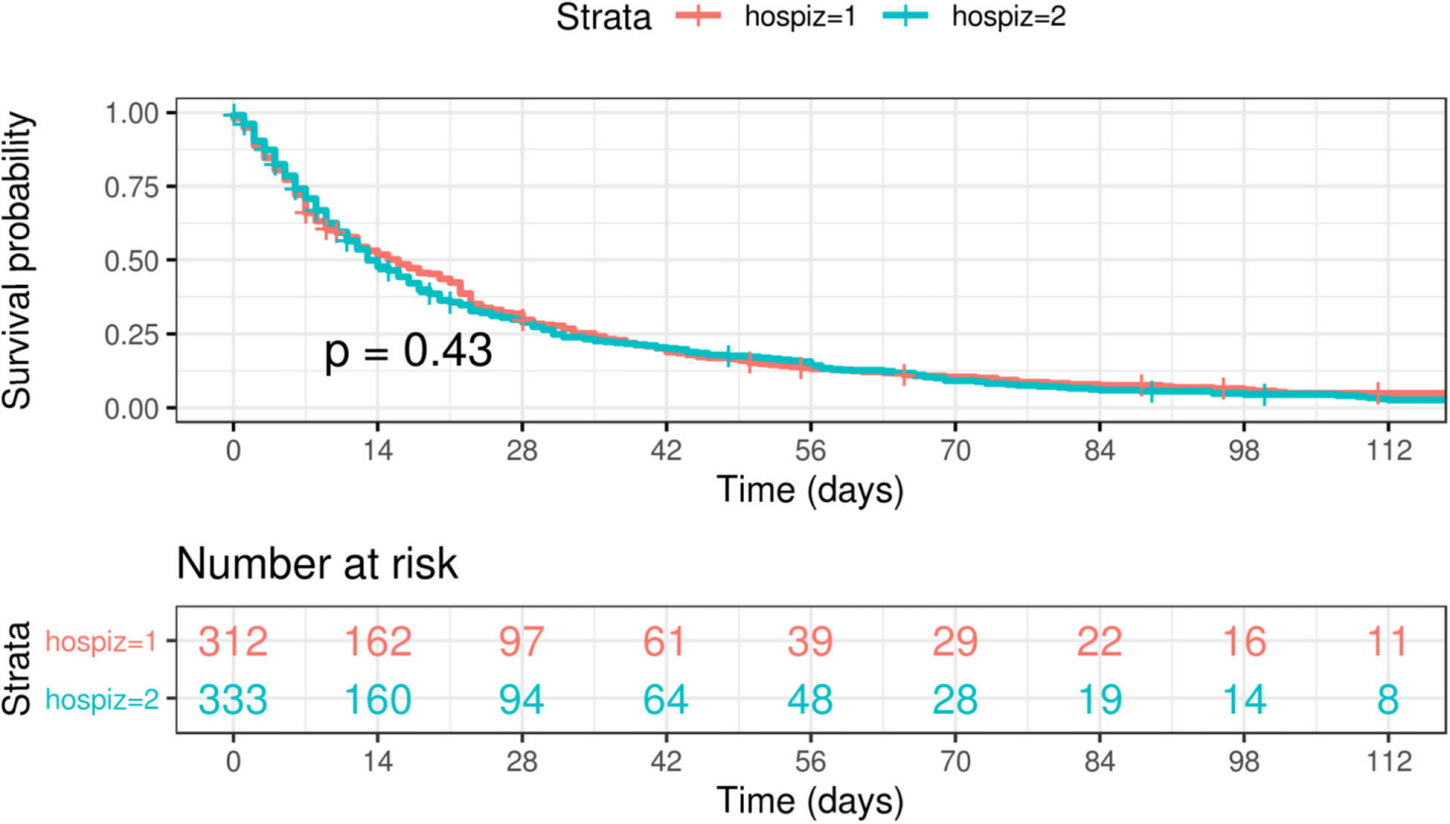
Survival time for all patients with malignant disease

A total of 706 patients were analysed (Table 1); 645 (91%) suffered from a malignant disease [H1, 312 (93%) of 336 residents; H2, 333 (90%) of 370 residents]. In both hospices the number of women predominated [but no significant difference (*p* = 0.18)], and the average age was 72 years (*p* = 0.98). The course of the disease was similar in both hospices: the average residence time was 30 days in H1 and 26 days in H2 (*p* = 0.66) with a range from zero days to a maximum of 440 days. Only a small number of patients were discharged from the hospice (4% each, *p* = 1.00) or transferred to a hospital (1% each, *p* = 0.69); the majority died within the first 8 weeks after being admitted to the hospice (Fig. 1: H1, 87.5%; H2, 85.6%, *p* = 0.43).

**Table 1:** General overview: Patients with malignant primary disease in two hospices

Category and related ***p***-value^a^	Hospice 1 (H1)		Hospice 2 (H2)		Total	
**Total number of patients**	336	100%	370	100%	706	100%
**Patients with malignant disease**	312	93%	333	90%	645	91%
**Evaluation: Patients with a malignant disease**						
**Age in years (median,** ***p*** **= 0.98)**	72 (range: 33–96)		72 (range: 42–101)		72 (range: 33–101)	
**Residence time in days (*p*** **= 0.66)**	30 (range: 0–440)		26 (range: 0–335)		28 (range: 0–440)	
**Gender (*p*** **= 0.18)**						
Male	141	45%	132	40%	273	42%
Female	171	55%	201	60%	372	58%
**Malignant disease (primary diagnosis)**						
Bronchial carcinomas (*p* = 0.92)	56	18%	61	18%	117	18%
Gastrointestinal cancers (*p* = 0.67)	97	31%	98	29%	195	30%
Gynecological cancers (*p* = 0.03)	47	15%	73	22%	120	19%
Brain cancers (*p* = 0.49)	25	8%	32	10%	57	9%
ENT tract cancers (*p* = 0.57)	12	4%	16	5%	28	4%
Other cancer (bone cancers, thyroid carcinomas, skin malignomas, cancer of unknown pirmary; *p* = 0.72)	14	5%	18	5%	32	5%
Urological cancers (*p* = 0.78)	30	10%	29	9%	59	9%
Haematological systemic diseases (*p* < 0.01)	31	10%	6	2%	37	6%
**Clinical course**						
Discharge (*p* = 1.00)	13	4%	13	4%	26	4%
Hospitalization (*p* = 0.69)	2	1%	4	1%	6	1%
For survival time see Fig. 1						

^a^ Refers to the comparison of the two hospices

Among the malignant diseases, gastrointestinal cancers, gynaecological cancers and bronchial carcinomas were most common in both hospices. There was no significant difference in the spread of malignant diseases in both hospices, except haematological systemic diseases that were more common in H1 (*p* < 0.01) and gynecological cancers that were more common in H2 (*p* = 0.03) (Table 1).

### Patients in oncological therapy

A total of 39 of 645 patients (6%) with an underlying malignant disease underwent symptom-focused oncological therapy (Table 2). The odds of receiving an oncological therapy were significantly higher in H1 [H1, 33 patients (11%); H2, 6 patients (2%), *p* < 0.01]. The average age was similar in both hospices (H1, 69 years; H2, 68 years), but slightly lower than all patients with a malignant disease (H1 and H2, 72 years respectively); however, there was no significant difference (*p* = 0.25). The residence time for patients with a symptom-focused oncological therapy was longer in H1 (67 days) than in H2 (44 days). The odds of being discharged from the hospice were significantly lower (*p* = 0.05) and the length of stay was significantly longer for patients who received an oncological therapy compared to patients who did not. (*p* < 0.01). The number of women predominated in both hospices.

**Table 2:** Patients with malignant primary disease and oncological therapy in two hospices

Category and related p-value^a^	Hospice 1 (H1)		Hospice 2 (H2)		Total	
**Patients with oncological therapy in a hospice (*p*** **< 0.01)**	33	11%	6	2%	39	6%
**Age in years (average)**	69 (range: 44–93)		68 (range: 56–87)		68 (range: 44–93)	
**Residence time in days**	67 (range: 4–440)		44 (range: 4–100)		55 (range: 4–440)	
**Gender**						
Male	11	33%	1	17%	12	31%
Female	22	67%	5	83%	27	69%
**Malignant disease (primary diagnosis)**						
Bronchial carcinomas	5	15%	1	17%	6	15%
Gastrointestinal cancers	7	21%	0	0%	7	18%
Gynecological cancers	10	30%	4	67%	14	36%
Brain cancers	1	3%	1	17%	2	5%
ENT tract cancers	1	3%	0	0%	1	3%
Other cancer (bone cancers, thyroid carcinomas, skin malignomas, cancer of unknown pirmary)	0	0%	0	0%	0	0%
Urological cancers	6	18%	0	0%	6	15%
Hematological systemic diseases	3	9%	0	0%	3	8%
**Indication of oncological therapies (multiple indications per patient, where applicable)**	41		6		47	
Dyspnea due to anaemia (*p* = 0.58)	10	24%	0	0%	10	21%
Diarrhea due to cancer disease (p = 1.00)	2	5%	0	0%	2	4%
Bone pain (*p* = 0.65)	13	32%	2	33%	15	32%
Meningeal cancer manifestation (*p* = 1.00)	1	2%	0	0%	1	2%
Continuation of ongoing anti-proliferative therapy (p = 0.01)	0	0%	2	33%	2	4%
Nausea/vomiting due to cancer disease (p = 1.00)	1	2%	0	0%	1	2%
Complications due to leukocytosis (p = 1.00)	3	7%	0	0%	3	6%
Pain therapy (*p* = 0.12)	4	10%	2	33%	6	13%
Other complications due to cancer disease (p = 1.00)	3	7%	0	0%	3	6%
**Oncological therapy (multiple treatment, where applicable)**	41		6		47	
Anti-hormonal therapy (*p* = 0.27)	5	12%	2	33%	7	15%
Targeted therapies (antibody therapy, tyrosine kinase inhibitors, somatostatin analogues; *p* = 0.44)	4	10%	2	33%	6	13%
Chemotherapy (p = 0.01)	6	15%	0	0%	6	15%
Radiotherapy (*p* = 0.01)	6	15%	0	0%	6	13%
Bisphosphonates (p = 0.02)	10	24%	2	33%	12	26%
Transfusions (p < 0.01)	10	24%	0	0%	10	21%
**Clinical course**						
Discharge	2	6%	1	17%	3	8%
Hospitalization	1	3%	1	17%	2	5%
For survival time see Fig. 2						

^a^ Refers to the comparison of the two hospices

In both hospices, the distribution of underlying malignant diseases among patients receiving oncological therapy (Table 2) was similar to the distribution among all the examined patients with a malignant disease (Table 1).

**Fig. 2:**
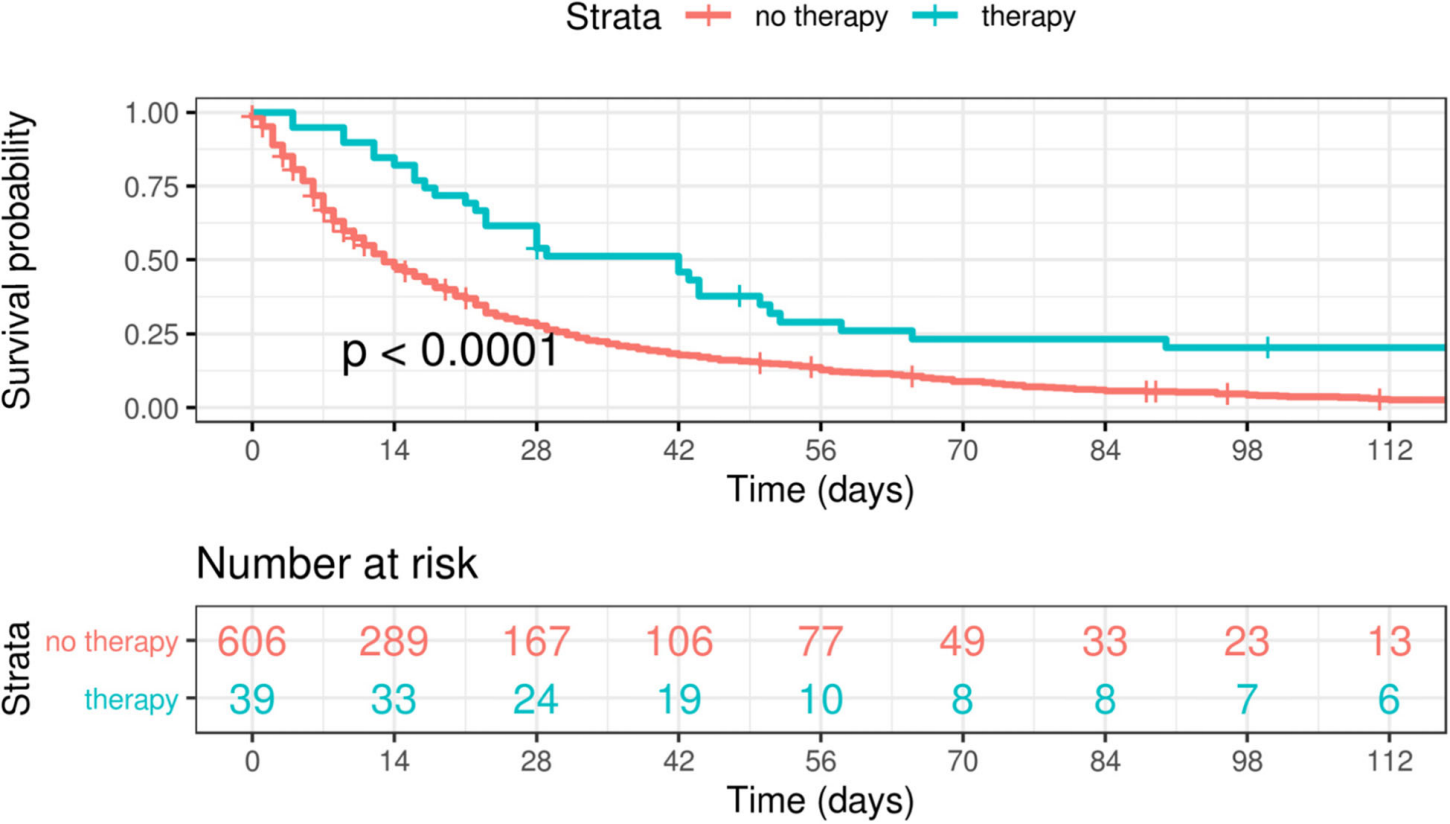
Survival time for all patients with oncological therapy

We analyzed survival times, i.e. the number of days from admission to death, and obtained that the hazard rate of patients who underwent symptom-focused oncological therapy is significantly different from those without therapy (Fig. 2 and Table 3), even if we control for hospice, age, sex and malignant disease (HR 0.49; *p* < 0.01). This suggests a lower instantaneous risk of mortality for patients who receive a symptom-focused oncological therapy. The hazard ratios for age (HR 1.01; *p* = 0.01), gender (HR 0.82; *p* = 0.04), brain cancer (HR 0.62; *p* = 0.005) and bisphosphonates (HR 0.48; *p* = 0.03; Table 4) were significantly different from 1 as well, which indicates that young and female patients and patients with bisphosphonates or without brain cancer have a lower instantaneous risk of mortality. Note that the statistical power is very low for the other therapies due to the small number of patients so that we cannot conclude that the other therapies do not have any relevant effect.

**Table 3:** Cox regression results for oncological therapy

	Hazard Ratio	95% Confidence Interval	***p***
Oncological therapy	0.49	[0.34,0.71]	0.0001
Hospice	1.03	[0.87,1.21]	0.7658
Age	1.01	[1,1.02]	0.0115
Sex (female)	0.82	[0.68,0.99]	0.0412
Gastrointestinal cancer	0.9	[0.71,1.14]	0.3987
Gynaecological cancer	0.83	[0.63,1.1]	0.191
Haematological systemic disease	1.41	[0.95,2.1]	0.0865
Brain cancer	0.62	[0.45,0.87]	0.0052
ENT tract cancer	0.69	[0.45,1.05]	0.0808
Other cancer	0.73	[0.49,1.11]	0.1426
Urological cancer	0.92	[0.66,1.29]	0.6391

**Table 4:** Cox regression results for the type of oncological therapy

	Hazard Ratio	95% Confidence Interval	***p***
Hospice	1.02	[0.86,1.2]	0.8329
Age	1.01	[1,1.02]	0.0212
Sex (female)	0.82	[0.68,0.99]	0.0416
Gastrointestinal cancer	0.92	[0.72,1.17]	0.4854
Gynaecological cancer	0.86	[0.65,1.13]	0.2805
Haematological systemic disease	1.38	[0.93,2.07]	0.1133
Brain cancer	0.62	[0.45,0.87]	0.0053
ENT tract cancer	0.69	[0.45,1.06]	0.0873
Other cancer	0.75	[0.49,1.13]	0.1626
Urological cancer	0.94	[0.67,1.32]	0.7376
Anti-hormonal therapy	0.47	[0.18,1.18]	0.1064
Radiotherapy	0.54	[0.22,1.36]	0.1929
Bisphosphonates	0.48	[0.25,0.91]	0.0254
Chemotherapy	1.49	[0.62,3.62]	0.3751
Transfusions	0.5	[0.24,1.02]	0.0564
Targeted therapies	0.41	[0.15,1.1]	0.0771

The most common indications for oncological therapy were dyspnea (H1, 24%; H2, 0%) and (bone) pain (H1, 42%; H2, 66%). Also the odds of receiving an oncological therapy were significantly higher for patients with dyspnea (*p* = 0.02) and bone pain (*p* = 0.01). In two patients in H2, anti-proliferative therapy was continued for reasons that could not be retrospectively detected (Table 2). When symptoms were treated, bone pain was the most common treatment indication for bronchial carcinomas and gynaecological and urological cancers. In case of gastrointestinal cancers and cancers of the ENT tract, dyspnoea due to anaemia was in the foreground, and in case of brain cancers and haematological systemic diseases, complications due to the malignant disease were the most important reasons for oncological therapy (Table 5). However, there are only low differences between the treatment indications in general because of the low number of cases.

**Table 5:** Indication and resulting oncological therapy in patients with a malignant primary disease in a hospice

Oncological disease	Indication oncological therapy (H1)	Oncological therapy^a^ (H1)	Indication oncological therapy (H2)	Oncological therapy^a^ (H2)
Bronchial carcinomas	Bone pain	Bisphosphonates (*n* = 3), Radiation (*n* = 1)	Continuation of ongoing therapy	Tyrosine kinase inhibitor (*n* = 1)
	Meningeal cancer manifestation	Intrathecal chemotherapy (*n* = 1)	none	none
	Dyspnea due to cancer disease	Intravenous chemotherapy (*n* = 1)	none	none
Gastrointestinal cancers	Bone pain	Bisphosphonates (n = 1)	none	none
	Dyspnea due to anaemia	Erythrocyte transfusion (*n* = 4)	none	none
	Diarrhea due to cancer disease	Somatostatin analogues (n = 2)	none	none
	Complications due to peritoneal carcinomatosis	Tyrosine kinase inhibitor (*n* = 1)	none	none
Gynecological cancers	Bone pain	Bisphosphonates (*n* = 4), Radiation (*n* = 1), Anti-hormonal therapy (*n* = 2)	Bone pain	Bisphosphonates (*n* = 2)
	Pain therapy	Anti-hormonal therapy (*n* = 2)	Pain therapy	Anti-hormonal therapy (*n* = 2)
	Dyspnea due to anaemia	Erythrocyte transfusion (*n* = 2)	none	none
	Nausea/vomiting due to cancer disease	Somatostatin analogues (*n* = 1)	none	none
Brain cancers	Complications due to brain cancer	Oral chemotherapy (*n* = 1) Radiation (*n* = 1)	Continuation of ongoing therapy	Tyrosine kinase inhibitor (*n* = 1)
ENT tract cancers	Dyspnea due to anaemia	Erythrocyte transfusion (*n* = 1)	none	none
Other cancer (bone cancers, thyroid carcinomas, skin malignomas, cancer of unknown pirmary)	none	none	none	none
Urological cancers	Bone pain	Bisphosphonates (*n* = 2), Radiation (*n* = 2)	none	none
	Dyspnea due to anaemia	Erythrocyte transfusion (*n* = 1)	none	none
	Pain therapy	Radiation (*n* = 1), Anti-hormonal therapy (*n* = 1)	none	none
Hematological systemic diseases	Complications due to leukocytosis	Oral/subcutaneous chemotherapy (*n* = 3)	none	none
	Dyspnea due to anaemia	Erythrocyte transfusion (*n* = 2)	none	none

^a^ multiple selections possible

Dyspnoea was primarily caused by anaemia, which was treated, only in H1, by the transfusion of erythrocyte concentrates (*p* < 0.01). Bisphosphonates and anti-hormonal treatments (for gynaecological and urological cancers) were used for pain therapy in both hospices, but bisphosphonates were used significantly more often in H1 (*p* = 0.02). In addition, radiotherapy and chemotherapy were only used in H1 (*p* = 0.01; Tables 2 and 5). Some of these therapies were used in combination. The symptomatic complications of malignant disease were only treated in H1 with anti-proliferative therapies (Table 5). Transfusions (*n* = 10, 21%), bisphosphonates (*n* = 12, 26%) and anti-hormonal therapies (*n* = 7, 15%) were the most common treatments for all patients with symptom-focused oncological treatment (Table 2).

## Discussion

Whether and how cancer therapies should be used in patients with a highly advanced haematological/oncological disease is controversial presently [14, 15, 20–23], particularly for the residents of a hospice [19, 24–27]. To obtain actual insight into the use of symptom-focused cancer therapies at the end of life, all residents in the two hospices in Lower Bavaria were retrospectively examined.

The capacity of 10 beds with patient care by general practitioners and palliative care physicians is common in German hospices [31, 32]. The integration of H1 into an ESMO-certified network is beyond this standard and probably has an impact on the type of patient care.

Of the 706 residents examined, 645 (91%) had a malignant disease; this predominance over non-cancer diseases is common in German hospices [32] and is reflected accordingly in the two hospices examined in this analysis. The average age of 72 years, slight predominance of women and average residence time of about 1 month were analogous to the general data of palliative and hospice patients in Germany [31–33]. The spread of the underlying malignant diseases, especially with regard to frequent cancer entities, was similar to the spread of cancer deaths in Germany [33]. This spread was approximately the same for both hospices. An exception were the haematological systemic diseases that were more frequently observed in H1 than in H2 (10% vs 2% of residents, *p* < 0.01). The integration of H1 into a network focusing on haematology and oncology is definitely an important factor. The conditions in H2 are more likely to reflect the reality in general care, especially because patients with a malignant haematological disease are under-represented in palliative care [34, 35] and the integration of a hospice into a haematological/oncological network is no standard in Germany.

Symptom-focused oncological therapies were used in both hospices, but only in a small number of patients (39 of 645 patients, 6%). However, a comparison of both hospices showed a significant difference in the number of therapies used (H1, 11%, *n* = 33; H2, 2%; *n* = 6; p < 0.01) for a comparable number of residents (H1, *n* = 312; H2, *n* = 333). This may be due to the medical care provided and organizational integration of the hospices; general practitioners and palliative care physicians provide independent care in H2, whereas haematologists and oncologists are additionally present in H1, providing a multidisciplinary approach. The medical specialization and the interdisciplinary approach might contribute to the choice of symptom-focused therapies [6, 14]. In addition, H1 is strongly integrated with outpatient and inpatient oncological therapy facilities, which minimizes the organizational effort for certain therapies (e.g. transfusions or radiation therapy).

In addition to the above mentioned structural factors, various patient factors also seem to have an influence on the choice of therapy. In both hospices, those patients who underwent symptom-focused oncological therapy lived significantly longer than those without oncological therapy - regardless of age, sex or malignant disease (HR 0.49, *p* < 0.01). Additionally patients with therapy had a significant prolonged stay in the hospice (p < 0.01) and significantly lower odds of being discharged (*p* = 0.05). However, it is unlikely that the symptom-focused oncological therapies are the cause of longer survival. Almost all of these therapies have no known life-prolonging effect, and in the statistical analysis, surprisingly, only the bisphosphonates had a significant influence. But there is no know effect of bisphosphonates that causes a better survival and therefore the data should not be overstated. Rather, a screening of patients in a more stable general condition seems to have taken place prior to the initiation of symptom-focused oncological therapy. The change from a strict “either/or” concept to a combined approach of hospice care and supportive cancer therapy has first application for a specific patient group here [25–27].

Specific cancer therapies were used in both hospices for symptom relief. The most common indications were dyspnea (21%) and (bone) pain (45%). These are typical and commonly occurring conditions in patients with advanced and incurable diseases [35, 36]. Palliative care approaches are generally used here [35, 36]; however, symptom-focused oncological therapies have already been successfully applied and should not be excluded a priori for hospice residents; rather they can represent meaningful symptom-oriented therapies [6–10]. This is also reflected in the fact that in our analysis patients with dyspnea (*p* = 0.02) or bone pain (*p* = 0.01) had a significant greater chance to receive a symptom-focused oncological therapy than patients without. Cancer-specific complications, such as bone pain in bronchial carcinomas, urological and gynaecological cancers or dyspnoea due to anaemia in gastrointestinal cancers (Table 5), are known symptoms of these diseases [37]. Due to the overall low number of cases, a clear distinction or a symptom allocation specific to the entity is only possible to a limited extent; the transitions are often seamless. But symptom-focused oncological therapies tend to be more symptom-oriented than cancer-specific.

Dyspnoea as a common symptom due to anaemia in cancer patients can be successfully treated by the use of erythrocyte transfusions [38]. Interestingly, dyspnoea was only documented in H1 and was treated by erythrocyte transfusions also only in H1 (H1, *n* = 10; H2, *n* = 0, *p* < 0.01). Due to the anticipated symptoms of patients in advanced stages, [35–37] it can be assumed that anaemia-related dyspnoea also occured in H2. However, the possibility of blood transfusion in a hospice is often quite complicated from an organizational point of view. In addition, the necessity of patients to undergo transfusion is often an obstacle for admission to a hospice [9], but hospice patients may have a greater acceptability of transfusions than nurses [39]. H1 was able to solve these problems through its integration into a network focusing on haematology and oncology. The same applies to the difference in the use of radiotherapeutic interventions for pain relief (H1, *n* = 6; H2, n = 0, *p* = 0.01). Short-term radiotherapy can lead to a significant reduction in pain, save painkillers and improve the quality of life [8]. However, there are also large organizational barriers for a hospice, which can be solved through cooperation, as observed for H1. Bisphosphonates, a potential useful co-analgesic [11, 12] for painful bone metastases or rather useful in preventing bone pain [40], were used according to the required ESMO clinical practice guidelines [12] in both hospices. In terms of figures, the more oncology-oriented H1 significantly prevailed in the number of cases in whom bisphosphonates were used (*p* = 0.02). In palliative condition, the use of anti-proliferative drugs in patients is controversial with distinct advantages [13, 16, 41, 42] and disadvantages [20–22]. However, the use of chemotherapy as a symptom-controlling therapy seems to be beneficial for patients with a very advanced disease [41, 42] or even in hospices [13]. Chemotherapy can be used in hospices only in a subgroup of carefully screened and symptomatic patients. In our analysis, only a small number of patients underwent chemotherapy (13% patients undergoing oncological therapy, corresponding to 0.9% of all patients with oncological disease in the hospice). The structural prerequisites seem to be necessary here because chemotherapies were only used in H1 (*p* = 0.01).

### Limitations

The study has some limitations. The analysis was undertaken only at two hospices, with partially significant structural differences. The study-design was retrospective and not close to a randomized trial or a propensity-weighted comparison. Therefore, the data must be estimated under these restrictions. Because of the small number of patients with an oncological therapy, the statistical power is low for some of the statistical tests. A representative statement regarding the use of symptom-focused oncological therapies in hospices throughout Germany therefore cannot be made. The results mainly describe the use and indication of oncological therapies for hospice residents. No statement can be made regarding the resulting effect on the quality of life and on the burden of symptoms - especially for patients with an ECOG 3 or 4 - because this information could not be retrospectively recorded on the basis of the available data. For the same reason, no comment can be made on the general preferences of patients and their relatives regarding the use of symptom-focused oncological therapies in hospices.

## Conclusion

Despite the limitation mentioned above, it is apparent that the use of symptom-focused oncological therapies in hospices is possible and will be further conducted. The focus here is on improving the burden of symptoms and thus the quality of life via the therapies applied and not on prolonging life. Hence, cancer-directed therapies could sometimes be an important part of the best palliative strategy. However, the indication for the use of such a therapy could well be related to the training focus of the attending physicians and the integration of hospices into network structures. It seems reasonable to include haematologists/oncologists and radiation therapists next to non-oncologists/palliative physicians in the care of hospice patients, to achieve the best possible care for hospice residents. The strict distinction between hospice and oncological cares of patients with a far advanced and incurable disease should be abandoned in favour of a combined and parallel treatment. The best possible therapy options can therefore be available to patients at any time. Initial approaches for a combined care already seem to exist.

## Data Availability

The datasets used and/or analysed during the current study are available from the corresponding author on reasonable request.

## Abbreviations

ESMO: European Society for Medical Oncology
H1: Hospice 1
H2: Hospice 2
HR: Hazard Ratio

## References

[1] Temel JS, Greer JA, Muzikansky A (2010). Early palliative care for patients with metastatic non-small-cell lung cancer. N Engl J Med.

[2] Farbicka P, Nowicki A (2013). Palliative care in patients with lung cancer. Contemp Oncol (Pozn).

[3] Hui D, Bruera E (2016). Integrating palliative care into the trajectory of cancer care. Nat Rev Clin Oncol.

[4] Parikh R, Kirch R, Smith T (2013). Early specialty palliative care — translating data in oncology into practice. N Engl J Med.

[5] Howie L, Peppercorn J (2013). Early palliative care in cancer treatment: rationale, evidence and clinical implications. Ther Adv Med Oncol.

[6] Park K, Lee C, Tseng Y (2017). Palliative radiation therapy in the last 30 days of life: a systematic review. Radiother Oncol.

[7] Barthelemy N, Jansen N, Gennigens C (2012). Does radiotherapy have a role in end-of-life care?. Rev Med Liege.

[8] van Oorschot B, Schuler M, Simon A (2011). Patterns of care and course of symptoms in palliative radiotherapy: a multicenter pilot study analysis. Strahlenther Onkol.

[9] LeBlanc T, Egan P, Olszewski A (2018). Transfusion dependence, use of hospice services, and quality of end-of-life care in leukemia. Blood.

[10] Orme J, Still D, Day R (2013). The experiences of patients undergoing blood transfusion in a day hospice. Int J Palliat Nurs.

[11] Mitra R, Jones S (2012). Adjuvant analgesics in cancer pain: a review. Am J Hosp Palliat Care.

[12] Fallon M, Giusti R, Aielli F (2018). Management of cancer pain in adult patients: ESMO clinical practice guidelines. Ann Oncol.

[13] Schonwetter R, Roscoe L, Nwosu M (2006). Quality of life and symptom control in hospice patients with cancer receiving chemotherapy. J Palliat Med.

[14] Haverhals L, Manheim C, Mor V (2019). The experience of providing hospice care concurrent with cancer treatment in the VA. Support Care Cancer.

[15] Mor V, Joyce N, Coté D (2016). The rise of concurrent care for veterans with advanced cancer at the end of life. Cancer.

[16] Mor V, Wagner T, Levy C (2019). Association of Expanded VA hospice care with aggressive care and cost for veterans with advanced lung Cancer. JAMA Oncol.

[17] Loriot Y, Miller K, Sternberg C (2015). Effect of enzalutamide on health-related quality of life, pain, and skeletal-related events in asymptomatic and minimally symptomatic, chemotherapy-naive patients with metastatic castration-resistant prostate cancer (PREVAIL): results from a randomised, phase 3 trial. Lancet Oncol.

[18] Tombal B, Saad F, Penson D (2019). Patient-reported outcomes following enzalutamide or placebo in men with non-metastatic, castration-resistant prostate cancer (PROSPER): a multicentre, randomised, double-blind, phase 3 trial. Lancet Oncol.

[19] van Oorschot B (2018). Krebs im Endstadium: Überengagierte Versorgung am Lebensende. Dtsch Arztebl.

[20] Prigerson H, Bao Y, Shah M (2015). Chemotherapy use, performance status, and quality of life at the end of life. JAMA Oncol.

[21] Zhang B, Nilsson M, Prigerson H (2012). Factors important to patients’ quality of life at the end of life. Arch Intern Med.

[22] Wu C, Hsu T, Chang C (2016). Palliative chemotherapy affects aggressiveness of end-of-life care. Oncologist.

[23] Kaur J, Mohanti B (2011). Transition from curative to palliative Care in Cancer. Indian J Palliat Care.

[24] Casarett D, Fishman J, Lu H (2009). The terrible choice: re-evaluating hospice eligibility criteria for cancer. J Clin Oncol.

[25] Casarett D, Van Ness P, O'Leary J (2006). Are patient preferences for life-sustaining treatment really a barrier to hospice enrollment for older adults with serious illness?. J Am Geriatr Soc.

[26] Casarett D, Fishman J, O'Dwyer P (2008). How should we design supportive cancer care? The patient's perspective. J Clin Oncol.

[27] Balboni T (2019). Hospice and anticancer therapy-shifting from an either/or to a both/and treatment model. JAMA Oncol.

[28] Team RC (2018). R: A Language and Environment for Statistical Computing.

[29] Kassambara, Alboukadel, Kosinski. Survminer (2019). Drawing survival curves using ‘Ggplot2’.

[30] Therneau T (2015). A Package for Survival Analysis in S.

[31] Robert Koch Institute and Destatis (2015). Health in Germany. Health reporting by the federal government.

[32] Jansky M, Nauck F, Jasper B (2017). Gutachten zum Bedarf an Hospizbetten in Nordrhein-Westfalen.

[33] Cancer in Germany 2013/2014. 11. Edition: Robert Koch Institute and the Society of Epidemiological Cancer Registry in Germany. 2017 https://www.krebsdaten.de/Krebs/DE/Content/Publikationen/Krebs_in_Deutschland/kid_2017/krebs_in_deutschland_2017.pdf?__blob=publicationFile. Accessed 27 Oct 2019.

[34] Oechsle K (2019). Palliative Care in Patients with hematological malignancies. Oncol Res Treat.

[35] Kaiser F, Rudloff L, Vehling-Kaiser U (2017). Palliative home care for patients with advanced haematological malignancies-a multicenter survey. Ann Hematol.

[36] Schneider W, Eichner E, Thoms U (2015). Specialised out-patient palliative care (SAPV) in Bavaria: efficiency, structural and process-related effects and rural care. Gesundheitswesen.

[37] German Society for Hematology and Medical Oncology. 2019. https://www.onkopedia.com/de/onkopedia/guidelines. Accessed 27 Oct 2019.

[38] Mercadante S, Ferrera P, Villari P (2009). Effects of red blood cell transfusion on anemia-related symptoms in patients with cancer. J Palliat Med.

[39] Meystre C, Burley N, Ahmedzai S (1997). What investigations and procedures do patients in hospices want? Interview based survey of patients and their nurses. BMJ.

[40] Porta-Sales J, Garzón-Rodríguez C, Llorens-Torromé S (2017). Evidence on the analgesic role of bisphosphonates and denosumab in the treatment of pain due to bone metastases: a systematic review within the European Association for Palliative Care guidelines project. Palliat Med.

[41] Tannock I, Osoba D, Stockler M (1996). Chemotherapy with mitoxantrone plus prednisone or prednisone alone for symptomatic hormone-resistant prostate cancer: a Canadian randomized trial with palliative end points. J Clin Oncol.

[42] Lahoud M, Kourie H, Antoun J (2016). Road map for pain management in pancreatic cancer: a review. World J Gastrointest Oncol.

